# Pistachio shell powder may be included in diets for weanling pigs without compromising growth performance or blood characteristics

**DOI:** 10.1093/tas/txag002

**Published:** 2026-01-06

**Authors:** Yeonwoo Kim, Su A Lee, Hans H Stein

**Affiliations:** Department of Animal Sciences, University of Illinois, Urbana, IL, 61801, United States; Department of Animal Sciences, University of Illinois, Urbana, IL, 61801, United States; Department of Animal Sciences, University of Illinois, Urbana, IL, 61801, United States

**Keywords:** alternative ingredients, blood urea nitrogen, dietary fiber, feed efficiency, pistachio shell powder, weanling pigs

## Abstract

An experiment was conducted to test the hypothesis that pistachio shell powder can be used as a fiber source in diets for weanling pigs without any negative impacts on pig growth performance. A total of 160 newly weaned pigs (initial body weight = 5.23 ± 0.54 kg) were allotted to 4 dietary treatments using a randomized complete block design with initial body weight being the blocking factor. There were 10 replicate pens per treatment. The control diet contained mainly corn and soybean meal and three additional diets were formulated to contain 5.0, 7.5, or 10.0% pistachio shell powder at the expense of corn. Dietary nutrient and energy concentrations were maintained among diets. Average daily gain (**ADG**), average daily feed intake (**ADFI**), and gain to feed ratio (**G:F**) were calculated for phase 1 (d 1 to 21), phase 2 (d 22 to 42), and for the overall experiment. Blood samples were collected at the end of each phase to analyze for blood characteristics. Fecal scores were also recorded during phase 1. Phase 1 results indicated that ADG, final body weight, and G:F tended to increase as pistachio shell powder increased in the diet (quadratic; *P < *0.10), and ADFI increased as pistachio shell powder increased in the diet (quadratic; *P < *0.05). In phase 2 and overall, ADG and G:F were not affected by treatment, but ADFI from day 1 to 42 tended to increase as the inclusion of pistachio shell powder increased in the diets (quadratic, *P < *0.10). On day 21, total protein in blood increased (quadratic, *P < *0.05) with increasing pistachio shell powder in diets. Albumin and gamma-glutamyl transferase also increased (linear, *P < *0.05) and chloride (linear, *P < *0.10) tended to increase whereas glutamate dehydrogenase and bicarbonate (linear, *P < *0.10) tended to decrease as pistachio shell powder increased in diets. On day 42, red blood cell count was reduced by pistachio shell powder (quadratic, *P < *0.05) whereas mean corpuscular hemoglobin tended to increase (linear, *P < *0.10), blood urea nitrogen increased (quadratic, *P < *0.05), chloride tended to increase (quadratic, *P < *0.10), and cholesterol increased (linear, *P < *0.05) with increasing dietary pistachio shell powder. In conclusion, with the exception of a tendency for increased ADFI, overall growth performance of weanling pigs was not affected by including up to 10% pistachio shell powder in diets for weanling pigs indicating that pistachio shell powder may be added to diets for weanling pigs.

## Teaser

Pistachio blanks are a high fiber co-product from the pistachio industry and may be included in diets for weanling pigs as a high-fiber ingredient.

## Introduction

In recent years, there has been a growing interest in using agricultural co-products as ingredients in diets for livestock including pigs (Zijlstra and Beltranena 2013). Pistachio shell powder is a high-fiber co-product from the pistachio nut industry that may be used in diets for gestating sows (Kim et al. 2024). With increasing global demand for pistachios, the production of pistachio shells has also increased, resulting in a greater supply of this co-product. Using pistachio shell powder that otherwise would be discarded as a fiber ingredient in animal diets may also improve sustainability of animal production and reduce the environmental impact of animal feed production, and thus, contribute to more sustainable and circular production methods.

Results of recent research demonstrated that gestating sows have high fermentability of fiber in pistachio shell powder (Kim et al. 2024), but there are no data to document effects of using pistachio shell powder in diets for weanling pigs. The transition from a milk-based to a solid diet during the post-weaning period frequently causes intestinal distress and poor growth performance (Zheng et al. 2021). Dietary fiber supplementation may be beneficial during this period because gastrointestinal motility may be increased and microbial fermentation may be enhanced, which reduces intestinal pH (Stein and Kil 2006; Chen et al. 2020). Insoluble fiber is effective in improving growth performance during the initial two weeks after weaning, which is most likely due to their ability to increase diet and intestinal bulking and improve gastrointestinal function (Chen et al. 2020). Inclusion of dietary fiber in diets fed to weanling pigs may reduce diarrhea (Mateos et al. 2006), which further indicates that fiber may positively impact intestinal health. Because pistachio shell powder mostly contains insoluble dietary fiber, inclusion in diets for weanling pigs may increase growth performance and reduce diarrhea during the post-weaning phase. Use of 3 or 6% almond hulls in diets for weanling or growing pigs tended to increase growth performance (Ahammad et al. 2024a, 2024b), but no data documenting the impact of pistachio shell powder on growth performance and health of weaning pigs have been reported. Inclusion of dietary fiber in diets for pigs usually reduce energy and nutrient digestibility (Rodriguez et al. 2020) because of the reduced ability of pigs to ferment dietary fiber compared with digestion of starch and other nutrients, although that is not always the case (Mateos et al. 2006; Ahammad et al. 2024a, 2024b). However, because of the lack of data it is not known if pistachio shell powder will positively or negatively impact growth of weaned pigs. Therefore, an experiment was conducted to test the hypothesis that pistachio shell powder can be used as a fiber source in diets for weanling pigs to reduce post-weaning diarrhea without compromising growth performance and blood characteristics.

## Materials and methods

All animal procedures were approved by the Institutional Animal Care and Use Committee at the University of Illinois, Urbana, IL, USA, before the experiment was initiated (approval number: 24180). Pigs were the offspring of Line 800 males mated to Camborough females (Pig Improvement Company, Henderson, TN, USA). Pigs were farrowed at the University of Illinois Swine Research Center (Champaign, IL, USA) and weaned at approximately 20 days of age. At weaning, pigs were moved to a nursery facility and allotted to experimental diets that were fed immediately after weaning. Pistachio shell powder was procured from The Wonderful Company (Los Angeles, CA, USA) and ground to an average particle size of approximately 450 microns. Whey powder was procured from Prairie Farms Dairy Inc. (Edwardsville, IL, USA) and soybean meal was procured from Solae LLC (Gibson City, IL, USA). Locally grown corn was obtained from the University of Illinois Feed Mill (Urbana, IL, USA), and spray-dried plasma (APC LLC, Ankeny, IA, USA) was also included in phase 1 diets (Table 1). The same batches of all ingredients were used in all diets (Table 2).

**Table 1.** Analyzed nutrient composition of feed ingredients, as-is basis.

Item, %	Corn	Soybean meal	Pistachio shell powder	Spray dried plasma	Whey powder
**Dry matter**	86.82	88.72	96.91	90.53	89.89
**Ash**	1.42	6.26	4.81	7.36	7.21
**Crude protein**	7.57	45.65	3.11	80.12	11.53
**Acid hydrolyzed ether extract**	3.32	2.21	1.62	0.33	…
**Gross energy, kcal/kg**	3,836	4,119	4,523	4,827	3,641
**Total dietary fiber**	12.90	20.50	87.60	…	…
** Soluble dietary fiber**	2.10	3.10	1.20	…	…
** Insoluble dietary fiber**	10.80	17.40	86.40	…	…
**Indispensable amino acids**					
** Arginine**	0.37	3.30	0.27	4.51	0.25
** Histidine**	0.24	1.26	0.07	2.24	0.23
** Isoleucine**	0.31	2.29	0.13	2.17	0.62
** Leucine**	1.02	3.64	0.22	7.11	1.08
** Lysine**	0.27	2.91	0.17	6.23	0.91
** Methionine**	0.16	0.62	0.04	0.82	0.18
** Phenylalanine**	0.41	2.43	0.15	4.42	0.34
** Threonine**	0.29	1.77	0.11	5.12	0.69
** Tryptophan**	0.05	0.49	0.03	1.03	0.20
** Valine**	0.39	2.36	0.18	4.09	0.62
** Total**	3.51	21.07	1.37	37.74	5.12
**Dispensable amino acids**					
** Alanine**	0.61	2.01	0.15	3.89	0.52
** Aspartic acid**	0.58	5.22	0.29	6.93	1.15
** Cysteine**	0.17	0.62	0.06	2.59	0.26
** Glutamic acid**	0.60	8.55	0.62	10.08	1.95
** Glycine**	0.30	1.93	0.14	2.77	0.22
** Proline**	0.73	2.31	0.13	4.01	0.67
** Serine**	0.37	1.94	0.16	4.75	0.52
** Tyrosine**	0.25	1.71	0.05	3.82	0.25
** Total**	3.61	24.29	1.60	38.84	5.54
**Total amino acids**	7.12	45.36	2.97	76.54	10.66

**Table 2.** Ingredient and nutrient composition of diets, as fed basis.

Item	Phase 1 (6–11 kg)				Phase 2 (11–25 kg)			
	Control	5.0%	7.5%	10.0%	Control	5.0%	7.5%	10.0%
**Ingredients, %**								
** Ground corn**	40.77	35.78	32.87	28.33	63.78	57.84	53.28	49.91
** Soybean meal, dehulled, 46% crude protein**	28.00	27.00	27.00	27.85	30.77	30.80	31.66	32.14
** Pistachio shell powder**	…	5.00	7.50	10.00	…	5.00	7.50	10.00
** Protein plasma, spray dried**	3.00	3.00	3.00	3.00	…	…	…	…
** Soybean oil**	2.20	3.06	3.45	4.66	2.00	2.85	4.05	4.46
** Whey powder**	23.00	23.00	23.00	23.00	…	…	…	…
**_l_-Lysine HCl**	0.27	0.31	0.32	0.30	0.39	0.40	0.38	0.37
**_dl_-Methionine**	0.14	0.17	0.18	0.18	0.13	0.15	0.15	0.16
**_l_-Threonine**	0.04	0.07	0.07	0.07	0.11	0.12	0.13	0.12
** Dicalcium phosphate**	0.63	0.69	0.70	0.71	1.11	1.14	1.15	1.16
** Limestone**	1.05	1.02	1.01	1.00	0.81	0.80	0.80	0.78
** Vitamin-mineral premix^a^**	0.50	0.50	0.50	0.50	0.50	0.50	0.50	0.50
** Salt**	0.40	0.40	0.40	0.40	0.40	0.40	0.40	0.40
**Analyzed composition**								
**Gross energy, kcal/kg**	3965	3972	3946	3969	3871	3888	3791	3729
**Dry matter, %**	89.24	88.75	88.54	87.70	87.35	86.87	86.04	85.82
**Crude protein, %**	21.52	19.97	19.51	21.33	20.26	20.73	19.42	19.24
**Total dietary fiber, %**	10.10	14.54	15.99	18.88	14.21	18.10	19.83	21.82
**Indispensable amino acids**								
** Arginine**	1.25	1.14	1.12	1.25	1.27	1.22	1.20	1.20
** Histidine**	0.57	0.52	0.51	0.56	0.55	0.53	0.52	0.51
** Isoleucine**	1.00	0.93	0.91	0.99	0.88	0.85	0.84	0.81
** Leucine**	1.91	1.80	1.72	1.86	1.73	1.69	1.64	1.59
** Lysine**	1.52	1.44	1.44	1.60	1.46	1.40	1.36	1.34
** Methionine**	0.42	0.41	0.38	0.44	0.43	0.40	0.38	0.43
** Phenylalanine**	1.03	0.96	0.93	1.03	1.01	0.98	0.96	0.93
** Threonine**	0.97	0.91	0.90	0.99	0.88	0.85	0.80	0.77
** Tryptophan**	0.23	0.22	0.22	0.23	0.21	0.22	0.21	0.22
** Valine**	1.13	1.06	1.04	1.14	0.99	0.96	0.94	0.91
** Total**	10.03	9.39	9.17	10.09	9.41	9.10	8.85	8.71
**Dispensable amino acids**								
** Alanine**	1.06	0.99	0.96	1.02	1.01	0.98	0.95	0.93
** Aspartic acid**	2.20	2.02	1.99	2.19	2.01	1.95	1.91	1.86
** Cysteine**	0.39	0.36	0.35	0.38	0.31	0.30	0.28	0.27
** Glutamic acid**	3.81	3.55	3.43	3.74	3.68	3.55	3.50	3.37
** Glycine**	0.79	0.73	0.72	0.79	0.83	0.80	0.78	0.77
** Proline**	1.21	1.13	1.09	1.15	1.18	1.13	1.11	1.07
** Serine**	0.93	0.86	0.84	0.91	0.87	0.84	0.82	0.80
** Tyrosine**	0.72	0.65	0.63	0.68	0.68	0.66	0.62	0.60
** Total**	11.11	10.29	10.01	10.86	10.57	10.21	9.97	9.67
**Total amino acids**	21.14	19.68	19.18	20.95	19.98	19.31	18.82	18.38

a Provided the following quantities of vitamins and micro-minerals per kilogram of complete diet: Vitamin A as retinyl acetate, 11,136 IU; vitamin D_3_ as cholecalciferol, 2208 IU; vitamin E as _dl_-alpha tocopheryl acetate, 66 IU; vitamin K as menadione dimethylprimidinol bisulfite, 1.42 mg; thiamin as thiamine mononitrate, 0.24 mg; riboflavin, 6.59 mg; pyridoxine as pyridoxine hydrochloride, 0.24 mg; vitamin B_12_, 0.03 mg; _d_-pantothenic acid as _d_-calcium pantothenate, 23.5 mg; niacin, 44.1 mg; folic acid, 1.59 mg; biotin, 0.44 mg; Cu, 20 mg as copper chloride; Fe, 126 mg as ferrous sulfate; I, 1.26 mg as ethylenediamine dihydriodide; Mn, 60.2 mg as manganese hydroxychloride; Se, 0.3 mg as sodium selenite and selenium yeast; and Zn, 125.1 mg as zinc hydroxychloride.

### Diets, animals, and experimental design

A two-phase feeding program was used, with day 1 to 21 as phase 1 and day 22 to 42 as phase 2. A total of 160 newly weaned pigs with an initial body weight of 5.23 ± 0.54 kg were used in a randomized complete block design with 10 blocks of 16 pigs per block. Within each block, pigs were randomly assigned to 4 dietary treatments using the Experimental Animal Allotment Program (Kim and Lindemann 2007), and for each treatment, there were 10 replicate pens with 2 barrows and 2 gilts in each pen. Initial body weight was the blocking factor. A control diet based on corn and soybean meal was formulated, and 3 additional diets were formulated by including 5.0, 7.5, or 10.0% pistachio shell powder at the expense of corn. All diets in both phases were formulated to meet current nutrient requirements for weanling pigs (NRC 2012) and inclusion of crystalline amino acids was increased as pistachio shell powder was increased in the diets. Soybean oil inclusion was also adjusted as pistachio shell powder inclusion increased to maintain diets isocaloric.

Pigs were housed in pens (1.2 × 1.4 m) in an environmentally controlled barn. Floors were fully slatted with a plastic coating. A 4-hole feeder and a nipple drinker were installed in each pen. Temperature, humidity, lighting, feeder and water space were identical for all pens. The barn had a negative pressure ventilation system and lights were turned on at all times. Barn temperatures were 30°C in week 1 post-weaning, 28°C in week 2, 26°C in week 3, 24°C in week 4, and 22°C in weeks 5 and 6 post-weaning. Municipal water for human consumption was sourced from the city of Champaign (IL, USA).

### Sample and data collection

Individual pig weights were recorded at the beginning of the experiment, on day 21, and at the end of the 42-day experiment. Daily feed allotments were recorded and the weights of feed left in feeders were recorded on day 21 and on the last day of the experiment to calculate feed consumption. Fecal scores were assessed visually per pen every other day during phase 1 using a score from 1 to 5 (1: firm stool; 2: normal firm stool; 3: moderately loose stool; 4: loose and watery stool; and 5: very watery stool).

On day 1, 21, and 42, two blood samples were collected from the jugular vein of the pig in each pen whose body weight on day 1 was closest to the pen average using a 20-gauge needle. Pigs were not fasted prior to blood collection. The same pig was bled on days, 1, 21 and 42. One blood sample was collected in a heparinized vacutainer, and the other blood sample was collected in a vacutainer containing ethylenediaminetetraacetic acid. Blood samples in vacutainers containing ethylenediaminetetraacetic acid were centrifuged at 4000× g at 4°C for 13 min, and plasma was recovered and stored at −20°C until analysis.

### Chemical analysis

All diet and ingredient samples were analyzed in duplicate for concentrations of gross energy using a bomb calorimeter (Model 6400, Parr Instruments, Moline, IL, USA), and nitrogen was analyzed by combustion (method 990.03; AOAC Int. 2019) using a LECO FP628 analyzer (LECO Corp., Saint Joseph, MI, USA) with the subsequent calculation of crude protein as nitrogen × 6.25. Dry matter was also analyzed in diet and ingredient samples by oven drying at 135°C for 2 h (method 930.15, AOAC Int. 2019) and ingredient samples were analyzed for dry ash (method 942.05; AOAC Int. 2019). All ingredient samples were analyzed for insoluble dietary fiber and soluble dietary fiber according to method 991.43 (AOAC Int. 2019) using the AnkomT^DF^ Dietary Fiber Analyzer (Ankom Technology, Macedon, NY, USA). Total dietary fiber was calculated as the sum of insoluble and soluble dietary fiber. All ingredient samples were also analyzed for acid hydrolyzed ether extract using the acid hydrolysis filter bag technique (Ankom HCl Hydrolysis System; Ankom Technology, Macedon, NY, USA) followed by crude fat extraction using petroleum ether (AnkomXT15 Extractor; Ankom Technology, Macedon, NY, USA). All diet and ingredient samples were analyzed for amino acids [method 982.30 E (a, b, c); AOAC Int. 2019] at the University of Missouri Experiment Station (Columbus, MO, USA). Heparinized plasma samples were analyzed for red blood cells, hemoglobin, mean cell volume, white blood cells, and albumin, whereas blood samples collected in vacutainers containing ethylenediaminetetraacetic were analyzed for other blood characteristics including chloride, sodium, cholesterol, and bicarbonate using a Beckman Coulter Clinical Chemistry AU analyzer (Beckman Coulter, AU680, Urbana, IL, USA).

### Calculations and statistical analysis

Data for growth performance were summarized at the conclusion of the experiment to calculate average daily gain (**ADG**), average daily feed intake (**ADFI**), and gain to feed ratio (**G:F**) for each pen and for each treatment group. Normality of residuals and homogeneity were verified using the UNIVARIATE procedure (SAS Institute Inc., Cary NC, USA ). Outliers were identified as values that deviated from the 1st or 3rd quartiles by more than 3 times the interquartile range using Internally Studentized Residuals (Tukey 1977). However, no outliers were detected. All data were analyzed using the PROC MIXED of SAS with the pen as the experimental unit. The model included diet as fixed effect and block as the random effect. Statistical significance was considered at *P < *0.05 and a tendency was considered at 0.05 ≤ *P < *0.10. A contrast statement was used to determine linear and quadratic effects of inclusion of pistachio shell powder in diets.

## Results

### Growth performance

Phase 1 results indicated that ADG and final body weight tended to increase as pistachio shell powder increased in the diet (quadratic, *P < *0.10; Table 3). The ADFI increased when pistachio shell powder was included at 5.0 or 7.5%, but if 10% was included, ADFI was not different from that obtained in pigs fed the control diet (quadratic, *P < *0.05). The G:F also tended to increase as inclusion of pistachio shell powder in diets increased (linear; *P < *0.10). Fecal score during phase 1 did not differ among treatments, and growth performance in phase 2 did also not differ among treatments. Overall ADG and G:F for the entire 42-day experiment were not impacted by dietary treatments, but overall ADFI tended to increase as inclusion of pistachio shell powder increased in the diet (quadratic, *P < *0.10).

**Table 3.** Growth performance of weanling pigs fed experimental diets^a^.

Item	Control	Pistachio shell powder (5.0%)	Pistachio shell powder (7.5%)	Pistachio shell powder (10.0%)	SEM	Linear	Quadratic
**Phase 1, d 1 to 21**							
** Initial body weight, kg**	5.09	5.24	5.24	5.23	0.19	0.134	0.342
** ADG^b^, g**	186.8	216.1	218.0	204.6	11.24	0.118	0.067
** ADFI^b^, g**	285.8	316.4	304.4	291.9	11.07	0.563	0.030
** G:F^b^, kg**	0.65	0.68	0.72	0.70	0.027	0.070	0.577
** Fecal score**	2.57	2.60	2.64	2.58	0.09	0.791	0.650
** Body weight (kg), day 21**	9.01	9.77	9.82	9.52	0.36	0.077	0.064
**Phase 2, d 22 to 42**							
** ADG, g**	696.1	732.5	681.6	700.9	21.45	0.852	0.446
** ADFI, g**	838.1	917.5	865.4	904.0	30.18	0.166	0.413
** G:F, kg**	0.84	0.80	0.79	0.78	0.033	0.168	0.761
** Body weight (kg), day 42**	23.63	25.16	24.13	24.24	0.64	0.501	0.138
**Overall, d 1 to 42**							
** ADG, g**	441.4	474.3	449.8	452.8	13.29	0.638	0.179
** ADFI, g**	562.0	612.3	580.1	587.6	17.07	0.251	0.082
** G:F, kg**	0.79	0.78	0.78	0.77	0.021	0.524	0.784

a Each least square mean represents 10 pens with 4 pigs per pen.

b ADG, average daily gain; ADFI, average daily feed intake; G:F, gain to feed ratio.

**Table 4.** Blood characteristics on d 21 of pig^a^.

Item	Control	Pistachio shell powder (5.0%)	Pistachio shell powder (7.5%)	Pistachio shell powder (10.0%)	SEM	Linear	Quadratic
**Red blood cell, ×10^6^/µL**	5.88	5.92	5.87	5.91	0.10	0.876	0.919
**Hemoglobin, g/dL**	10.08	10.23	10.15	10.14	0.19	0.858	0.479
**Hematocrit, %**	32.58	33.54	32.74	32.68	0.61	0.981	0.316
**Mean cell volume, fL^b^**	55.50	56.82	56.09	55.41	1.05	0.986	0.296
**Mean corpuscular hemoglobin, pg**	17.19	17.48	17.39	17.20	0.31	0.916	0.436
**Mean corpuscular hemoglobin concentration, g/dL**	30.97	30.77	30.97	31.14	0.26	0.648	0.397
**Platelets, ×10^3^/µL**	628	613	574	635	34	0.805	0.367
**Mean platelet volume, fL^b^**	10.37	10.92	10.96	10.57	0.18	0.212	0.019
**White blood cell, ×10^3^/µL**	17.34	19.90	17.55	18.42	0.92	0.558	0.190
**Segmental neutrophils, %**	39.20	45.44	34.11	38.27	2.62	0.366	0.280
**Lymphocytes, %**	53.86	45.61	57.69	53.61	2.85	0.592	0.179
**Mononucleosis, %**	5.06	4.16	6.07	5.46	0.87	0.412	0.494
**Eosinophils, %**	1.15	1.01	1.04	1.63	0.23	0.266	0.105
**Basophils, %**	0.52	0.60	1.12	0.65	0.21	0.300	0.422
**Absolute segmental neutrophils, ×10^3^/µL**	6.94	8.69	5.80	7.15	0.71	0.658	0.375
**Absolute band, ×10^3^/µL**	0.04	0.24	0.04	0.07	0.05	0.974	0.026
**Absolute lymphocytes, ×10^3^/µL**	9.22	8.78	10.08	9.80	0.68	0.373	0.631
**Absolute mononucleosis, ×10^3^/µL**	0.86	0.91	1.04	0.99	0.16	0.358	0.906
**Absolute eosinophils, ×10^3^/µL**	0.20	0.18	0.18	0.30	0.04	0.148	0.024
**Absolute basophils, ×10^3^/µL**	0.08	0.11	0.19	0.10	0.04	0.272	0.229
**Creatine, mg/dL**	0.59	0.59	0.60	0.58	0.02	0.870	0.629
**Blood urea nitrogen, mg/dL**	7.10	6.99	7.70	8.10	0.74	0.263	0.520
**Total protein, g/dL**	4.13	4.50	4.47	4.45	0.08	0.001	0.023
**Albumin, g/dL**	2.38	2.59	2.69	2.69	0.07	0.002	0.403
**Globulin, g/dL**	1.75	1.90	1.78	1.76	0.07	0.993	0.143
**Calcium, mg/dL**	11.08	10.56	10.93	10.80	0.30	0.605	0.470
**Phosphorus, mg/dL**	8.76	8.42	8.76	8.63	0.29	0.845	0.590
**Sodium, mmol/L**	141	141	141	141	0.57	0.345	0.584
**Chloride, mmol/L**	98	103	104	104	2.43	0.080	0.518
**Glucose, mg/dL**	111	108	111	109	2.94	0.768	0.753
**Alkaline phosphatase total, U/L^b^**	313	329	277	317	16.09	0.588	0.811
**Aspartate aminotransferase, U/L^b^**	30.30	25.23	29.25	33.70	2.69	0.407	0.046
**Gamma-glutamyl transferase, U/L^b^**	26.40	30.45	30.95	31.50	1.87	0.043	0.522
**Total bilirubin, mg/dL**	0.10	0.10	0.11	0.10	0.01	0.620	0.539
**Creatine phosphokinase, U/L^b^**	712	683	1245	956	257	0.268	0.969
**Cholesterol, mg/dL**	71	72	73	75	2.54	0.266	0.701
**Glutamate dehydrogenase, U/L^b^**	1.62	1.59	1.39	1.31	0.10	0.028	0.436
**Bicarbonate (total carbon dioxide), mmol/L**	28.05	27.07	24.30	26.80	0.92	0.069	0.203
**Magnesium, mg/dL**	1.91	1.87	1.97	1.91	0.05	0.705	0.919
**Triglycerides, mg/dL**	47.10	56.80	57.45	55.30	4.29	0.104	0.232
**Anion gap, mmol/L**	17.20	17.56	18.25	16.40	0.60	0.680	0.093

a Each least square mean represents 10 replicate pig.

b U/L, units per liter; fL, femtoliter.

**Table 5.** Blood characteristics of pig on day 42^a^.

Item	Control	Pistachio shell powder (5.0%)	Pistachio shell powder (7.5%)	Pistachio shell powder (10.0%)	SEM	Linear	Quadratic
**Red blood cell, ×10^6^/µL**	6.40	6.18	6.25	6.40	0.09	0.834	0.038
**Hemoglobin, g/dL**	11.23	11.27	11.42	11.50	0.20	0.296	0.547
**Hematocrit, %**	35.47	35.35	36.26	36.26	0.69	0.323	0.704
**Mean cell volume, fL^b^**	55.54	57.28	57.99	56.77	0.74	0.120	0.123
**Mean corpuscular hemoglobin, pg**	17.58	18.14	18.28	18.01	0.22	0.078	0.118
**Mean corpuscular hemoglobin concentration, g/dL**	31.65	31.62	31.52	31.72	0.25	0.975	0.658
**Platelets, ×10^3^/µL**	542	435	491	469	30	0.124	0.157
**Mean platelet volume, fL^2^**	8.84	10.56	9.51	9.99	0.79	0.170	0.198
**White blood cell, ×10^3^/µL**	17.87	18.03	18.21	15.80	1.09	0.290	0.218
**Segmental neutrophils, %**	27.40	26.76	23.42	24.17	2.61	0.228	0.957
**Lymphocytes, %**	62.81	64.16	67.41	66.16	2.58	0.516	0.845
**Mononucleosis, %**	7.09	5.57	6.84	7.04	1.01	0.971	0.312
**Eosinophils, %**	1.98	2.64	1.49	1.67	0.37	0.325	0.241
**Basophils, %**	0.59	0.87	0.87	0.98	0.19	0.135	0.795
**Absolute segmental neutrophils, ×10^3^/µL**	4.91	5.25	4.20	3.78	0.57	0.111	0.261
**Absolute band, ×10^3^/µL**	0.02	0.01	0	0.01	0.01	0.382	0.316
**Absolute lymphocytes, ×10^3^/µL**	10.54	11.66	12.31	10.51	0.99	0.728	0.201
**Absolute mononucleosis, ×10^3^/µL**	1.25	1.03	1.27	1.08	0.22	0.716	0.859
**Absolute eosinophils, ×10^3^/µL**	0.31	0.50	0.25	0.28	0.08	0.469	0.122
**Absolute basophils, ×10^3^/µL**	0.10	0.16	0.17	0.15	0.04	0.265	0.405
**Creatine, mg/dL**	0.73	0.70	0.79	0.76	0.04	0.312	0.798
**Blood urea nitrogen, mg/dL**	7.50	7.17	8.55	10.20	0.39	<0.001	0.001
**Total protein, g/dL**	4.86	4.82	5.15	5.06	0.14	0.165	0.784
**Albumin, g/dL**	3.20	3.19	3.36	3.43	0.11	0.114	0.471
**Globulin, g/dL**	1.66	1.64	1.79	1.63	0.07	0.847	0.483
**Calcium, mg/dL**	10.77	10.42	10.99	10.86	0.28	0.611	0.481
**Phosphorus, mg/dL**	12.59	9.29	9.83	10.15	1.58	0.241	0.312
**Sodium, mmol/L**	139	131	140	139	2.93	0.723	0.152
**Chloride, mmol/L**	101	95	101	101	2.11	0.786	0.085
**Glucose, mg/dL**	114	107	115	113	3.16	0.952	0.324
**Alkaline phosphatase total, U/L^b^**	237	231	234	246	13.85	0.692	0.461
**Aspartate aminotransferase, U/L^b^**	37.80	35.96	34.80	39.90	5.10	0.896	0.507
**Gamma-glutamyl transferase, U/L^b^**	27.95	28.28	29.10	29.70	1.92	0.498	0.836
**Total bilirubin, mg/dL**	0.11	0.11	0.10	0.11	0.01	0.486	0.663
**Creatine phosphokinase, U/L^b^**	1569	937	980	1276	375	0.409	0.202
**Cholesterol, mg/dL**	73	80	84	84	3.10	0.014	0.593
**Glutamate dehydrogenase, U/L^b^**	2.50	2.89	2.82	2.55	0.57	0.862	0.478
**Bicarbonate (total carbon dioxide), mmol/L**	27.25	26.11	27.20	27.80	0.88	0.640	0.240
**Magnesium, mg/dL**	1.93	1.84	1.98	1.97	0.06	0.385	0.203
**Triglycerides, mg/dL**	49.20	43.60	44.05	60.35	11.43	0.561	0.261
**Anion gap, mmol/L**	16.65	16.05	18.05	16.53	0.78	0.653	0.861

a Each least square mean represents 10 replicate pigs.

b U/L, units per liter; fL, femtoliter.

### Blood characteristics

No differences among treatments in blood characteristics on day 1 were observed (data not shown). Blood characteristics on day 21 indicated that mean platelet volume, absolute eosinophils and total protein increased as pistachio shell powder inclusion increased in the diet (quadratic, *P < *0.05; Table 4). Albumin, and gamma-glutamyl transferase also increased (linear, *P < *0.05) and chloride tended to increase as dietary pistachio shell powder increased in the diet (linear, *P < *0.10). In contrast, glutamate dehydrogenase decreased (linear, *P < *0.05) and bicarbonate tended to decrease (linear, *P < *0.10) with increasing dietary pistachio shell powder. Aspartate aminotransferase tended (quadratic, *P < *0.10) to decrease and then increase as the concentration of pistachio shell powder increased in diets.

On day 42, red blood cell counts decreased at 5.0 or 7.5% pistachio shell powder was included in the diet, but increased if 10% was used (quadratic, *P < *0.005; Table 5). Mean corpuscular hemoglobin tended to increase (linear, *P < *0.10), and blood urea nitrogen increased (linear and quadratic, *P < *0.001) as pistachio shell powder increased in the diet. Chloride tended to decrease (quadratic, *P < *0.10) if 5.0% pistachio shell powder was included in the diet, but if 7.5 or 10% was included, chloride did not change. Cholesterol also increased (linear, *P < *0.05) as pistachio shell powder increased in the diet.

## Discussion

Pistachio shell powder consists of ground pistachio shells and contains mostly insoluble dietary fiber (Kim et al. 2024). As the production of pistachios increases, more pistachio shells will become available as co-products. High-fiber co-products are often less valuable than corn and soybean meal because of the high concentration of dietary fiber, which is resistant to enzymatic hydrolysis in the small intestine, and therefore, cannot be digested by pigs (Anguita et al. 2006; Bindelle et al. 2008). Pigs can obtain energy from dietary fiber via microbial fermentation in the hindgut, which produces volatile fatty acids (Dierick et al. 1989; Macfarlane and Macfarlane 2003). However, younger pigs are less efficient at using dietary fiber than older pigs due to a less developed hindgut microbiome (Espinosa et al. 2020). Nevertheless, weanling pigs may benefit from dietary fiber because fiber can improve intestinal health and reduce the risk of diarrhea during the post-weaning period (Mateos et al. 2006; Jha et al. 2019). Beneficial effects of fiber in diets for weanling pigs may be due to increased synthesis of short chained fatty acids and subsequently reduced intestinal pH, which is detrimental to pathogen growth (Kil and Stein 2010). The analyzed nutrient composition of the ingredients used in the experiment were in agreement with expected values. Pistachio shell powder contained more than 80% insoluble dietary fiber, whereas corn and soybean meal have much less fiber, which is also in agreement with reported values (NRC 2012; Kim et al. 2024).

### Growth performance

The observation that the inclusion of pistachio shell powder in the diet did not affect overall growth performance indicated that under the conditions of this experiment, weanling pigs used up to 10% pistachio shell powder without any negative impacts on growth performance. This observation is in agreement with data indicating that inclusion of 3 or 6% almond hulls in diets for weanling pigs resulted in a tendency for improved average daily gain (Ahammad et al. 2024a). All diets were formulated to have identical concentrations of metabolizable energy and amino acids, which likely contributed to the lack of difference in growth performance among diets (Gutierrez et al. 2013; Jaworski et al. 2014). However, to maintain diets isocaloric, the inclusion of soybean oil increased as dietary pistachio shell powder increased, and results, therefore, reflect the combined effects of increased soybean oil and increased pistachio shell powder. Nevertheless, Ani et al. (2013) observed increased ADG in weanling pigs with inclusion of 10% soybean hulls in the diet, but reduced growth performance of weanling pigs fed diets containing soybean hulls has also been reported (Goehring et al. 2019).

Inclusion of 1.5 to 8% insoluble dietary fiber in diets for weaned pigs tends to increase ADFI and ADG (Flis et al. 2017). In the present experiment, 5% pistachio shell powder provided 4.3% insoluble dietary fiber to the diets, and 10% pistachio shell powder provided 8.6% total dietary fiber. The observation that ADG in phase 1 tended to increase as pistachio shell powder was added to the diets, therefore, is in agreement with the results by Flis et al. (2017), but it is not completely clear if there is a limit to the inclusion of total dietary fiber in diets for weanling pigs, and what that limit may be. However, because no reductions in ADFI were observed as pistachio shell powder was included in the diets, it is concluded that pigs did not have palatability issues with diets containing pistachio shell powder.

The observation that there were no differences in fecal scores during phase 1 among treatments was surprising because intestinal health may be improved if fiber is included in the diet (Mateos et al. 2006; Jha et al. 2019). However, pigs used in the experiment were of high health and diarrhea incidence was generally low regardless of dietary treatment, which may have been the reason no change was observed as pistachio shell powder was included in the diets. Nevertheless, because of the lack of differences in fecal scores among treatments, the hypothesis that pistachio shell powder may reduce diarrhea incidence in weanling pigs was rejected. Future experiments should explore the long-term effects of pistachio shell powder on metabolic health, gut microbiome composition, and nutrient digestibility to fully assess the utility of pistachio shell powder in commercial swine diets.

### Blood characteristics

Blood characteristics observed in this experiemnt remained within the normal physiological ranges for swine (Tumbleson and Kalish 1972) and were generally consistent with previous values (Casas and Stein 2016; Espinosa et al. 2017). The modest reduction in red blood cell count at intermediate inclusion levels of pistachio shell powder may indicate that moderate amounts of dietary fiber influence erythropoiesis or red blood cell turnover, whereas higher inclusion does not appear to have the same effect. Other red blood cell indices, including hemoglobin concentration, hematocrit, mean cell volume, and mean corpuscular hemoglobin concentration, were not influenced by treatments, indicating that the oxygen-carrying capacity was maintained regardless of the level of pistachio shell powder in the diets. The tendency for mean corpuscular hemoglobin to increase with greater levels of pistachio shell powder indicates that dietary fiber components enhance hemoglobin synthesis or iron utilization, resulting in greater hemoglobin content per red blood cell without altering cell size or number. This increase indicates that certain components of pistachio shell powder, possibly fermentable fiber, influence iron absorption and hemoglobin synthesis (Shah et al. 2009), thereby improving red blood cell hemoglobin content.

Platelet counts and mean platelet volume did not differ among dietary treatments, and all values were consistent with reference intervals for pre-weaning pigs (Faustini et al. 2003) indicating that pistachio shell powder did not affect platelet production or activation. Total protein and albumin concentrations were maintained within published normal ranges (Perri et al. 2017), indicating that protein nutrition and hepatic functions were likely uncompromised by the inclusion of pistachio shell powder in the diets. Bilirubin levels remained low and regardless of treatment were comparable to reference limits for total bilirubin in healthy pigs, which indicates that there was no hemolysis or liver dysfunction (Perri et al. 2017). The anion gap values stayed within reported normal levels, implying that acid–base balance was preserved despite dietary fiber inclusion (Cooper et al. 2014).

The increased cholesterol concentration that was observed as pistachio shell powder inclusion increased may be a result of altered lipid metabolism through the gut–liver axes influenced by bile acid dynamics and microbiota-mediated effects. As an example, fructo-oligosaccharides may reduce serum cholesterol by modulating bile acid profiles and microbial metabolism (Hu et al. 2023). Although pistachio shell powder does not have large concentrations of fructo-oligosaccharides (Kim et al. 2024), it is possible that other components of the fiber in pistachio shell powder influenced bile acid metabolism, but to our knowledge, no research has been conducted to confirm this hypothesis. Likewise, a possible impact of the intestinal microbiome on cholesterol synthesis has not been researched but may be an explanation for the increased cholesterol in pigs fed pistachio shell powder. The lack of changes in the concentration of triglyceride indicates that the effect may be specific to cholesterol regulation, rather than overall lipid absorption. It is, however, also possible that the increase in inclusion of soybean oil in diets with increased pistachio shell powder contributed to the increased cholesterol that was determined.

Blood urea nitrogen is a measure of the efficiency of amino acid utilization after absorption (Coma et al. 1995; Kohn et al. 2005). The observed linear increase in the concentration of blood urea nitrogen on day 42 in pigs fed diets containing pistachio shell powder indicates that high dietary fiber concentrations may reduce amino acid utilization efficiency in pigs, possibly as a result of increased needs for synthesis of diet-specific endogenous intestinal secretions. However, the observation that growth performance was not reduced as pistachio shell powder inclusion in diets increased does not indicate a reduced efficiency of amino acids except if diets contained more amino acids than required by the pigs, which is unlikely because diets were formulated to meet current requirements (NRC 2012). It is also possible that the increased blood urea nitrogen in pigs fed diets containing pistachio shell powder is a result of more nitrogen being needed in the hindgut for microbial protein synthesis as dietary fiber increased (Zervas and Zijlstra 2002). If that is correct, it is possible that some of the nitrogen that would otherwise have been excreted in the urine was used in the hindgut, and therefore, contributed to blood urea nitrogen, which would not have increased total nitrogen excretion from the pigs, and therefore not impacted growth performance. However, because we did not determine fecal nitrogen concentration or fecal bacterial protein, we cannot confirm that more microbial nitrogen was excreted from pigs fed the diets containing pistachio shell powder, but inclusion of almond hulls in diets for weanling or growing pigs did result in a tendency for reduced ammonia emissions, which may be a result of less nitrogen being excreted in the urine (Ahammad et al. 2024a, 2024b).

## Conclusion

Inclusion of up to 10% pistachio shell powder in diets for weanling pigs did not compromise overall growth performance, indicating that pistachio shell powder can be used as a partial replacement for conventional feed ingredients without negative effects on pig growth performance as long as diets are formulated to be isocaloric and meet al. nutrient requirements. Average daily feed intake is not reduced by pistachio shell powder indicating no negative effects on palatability. However, fecal scores were not reduced by inclusion of pistachio shell powder in the diets. The increase in cholesterol concentrations with greater pistachio shell powder inclusion may reflect alterations in lipid metabolism, potentially mediated by microbial fermentation and gut–liver interactions, but may also be related to increased soybean oil. Overall, pistachio shell powder may be included in diets for weanling pigs by up to 10%.

## Abbreviations:

ADFI: average daily feed intake
ADG: average daily gain
G:F: gain-to-feed ratio
